# Genome Sequence of Banana Streak MY Virus from the Pacific Ocean Island of Tonga

**DOI:** 10.1128/genomeA.00543-15

**Published:** 2015-05-28

**Authors:** Daisy Stainton, Mana’ia Halafihi, David A. Collings, Arvind Varsani

**Affiliations:** a Biomolecular Interaction Centre and School of Biological Sciences, University of Canterbury, Christchurch, New Zealand; b Ministry of Agriculture and Food, Forests and Fisheries of Tonga, Nuku-alofa, Kingdom of Tonga; c Department of Clinical Laboratory Sciences, Electron Microscope Unit, University of Cape Town, Rondebosch, Cape Town, South Africa; d Department of Plant Pathology and Emerging Pathogens Institute, University of Florida, Gainesville, Florida, USA

## Abstract

Banana streak disease is caused by a variety of banana-infecting badnaviruses. A genome of the episomal form of a banana streak MY virus was recovered from an infected banana plant sampled on Vava’u Island, Tonga, and shares >98% pairwise identity with the six other genomes available in public databases.

## GENOME ANNOUNCEMENT

Banana streak disease (BSD) is a disease of *Musa* spp. causing chlorotic streaks in leaves and yield reduction, with severe disease leading to the death of the plant. BSD is caused by multiple viral species of badnaviruses (genus *Badnavirus*, family *Caulimoviridae*). Badnaviruses have double-stranded DNA (dsDNA) genomes (~7.5 kb) encapsidated in bacilliform particles 30 × 130 nm in size (1, 2). Banana-infecting badnavirus genomes have three open reading frames (ORFs). ORF1 encodes a protein of unknown function, ORF2 encodes the virion-associated protein (3), and ORF3 encodes a 208-kDa polyprotein, which contains a movement protein, coat protein, aspartic protease, reverse transcriptase, and RNase H (4, 5).

There are currently four recognized banana-infecting badnavirus (BIB) species: banana streak GF virus (BSGFV), banana streak MY virus (BSMYV), banana streak OL virus (BSOLV), and banana streak VN virus (BSVNV), as well as a further six yet to be assigned a species status: banana streak CA virus (BSCAV), banana streak IM virus (BSIMV), banana streak UA virus (BSUAV), banana streak UL virus (BSULV), banana streak UM virus (BSUMV), and banana streak UI virus (BSUIV). BIBs exist in two forms, either the infectious episomal form with a circular double-stranded DNA genome, or the endogenous form in which the viral genome is integrated into the host genome. BIBs are able to be spread via infected propagules, and the episomal form is vectored by a number of mealy bug species, including *Dysmicoccus brevipes*, *Planococcus ficus*, and *Planococcus citri* (5–7). A number of endogenous BIBs are able to activate, resulting in the infective episomal form (8–12), which has been attributed to recent outbreaks (8). The genome sequences of 26 BIBs are available in public databases: BSGFV (*n* = 2), BSMYV (*n* = 6), BSOLV (*n* = 6), BSVNV (*n* = 3), BSCAV (*n* = 2), BSIMV (*n* = 3), BSUAV (*n* = 1), BSULV (*n* = 1), BSUMV (*n* = 1), and BSUIV (*n* = 1).

Total DNA was extracted from banana leaf samples (*n* = 134) collected from the islands of Vava’u, Tongatapu, and Ha’apai of Tonga in 2010 to 2011 using a GenCatch plant genomic DNA purification kit (Epoch Biolabs, USA). Using the screening primers MysF1/R1 (13), we identified the presence of BSMYV in 17 samples. Based on the sequence of the amplicon, back-to-back primers (Mys-B2B-F [5′ GAA GAA CAC AGA AGG GAA ATG GCT CG 3′] and Mys-B2B-R [5′-AGA GAT TCT GTT CCA CGC CGT TAA G- 3′]) were designed to recover the full genome using HiFi HotStart DNA polymerase (Kapa Biosystems, USA). We identified the episomal form of the virus (amplicon size, 7,650 nucleotides [nt]) in only one sample (TO213) from Vava’u Island. This amplicon was cloned into the pJET1.2 plasmid vector (Thermo Fisher Scientific, USA) and Sanger sequenced by primer walking at Macrogen, Inc. (South Korea). The genome of this Tongan BSMYV shares >98% genome-wide pairwise identity to the six BSMYV genomes available in GenBank that have been recovered from Australia (*n* = 1) (5), India (*n* = 4) (14), and an unknown location (*n* = 1) (15).

This report identifies the first episomal genome of BSMYV from Tonga.

### Nucleotide sequence accession number. 

The complete genome of BSMYV (isolate TO213) from Tonga has been deposited at GenBank under the accession no. KR014107.
